# Challenges in chemotherapy for head and neck cancer: A review

**DOI:** 10.6026/973206300210121

**Published:** 2025-02-28

**Authors:** Richa Wadhawan, Akansh Datta, Sita Gogula, Anand Krishnan, Dinesh Kumar Yadav, Tarun Choudhary

**Affiliations:** 1 Department of Oral Medicine, Diagnosis & Radiology, PDM Dental College & Research Institute, Bahadurgarh, Haryana, India; 2 Department of Oral and Maxillofacial Surgery, Teerthanker Mahaveer Dental College and Research Centre, Moradabad, Uttar Pradesh, India; 3 Department of Oral Medicine and Radiology, Lincoln University College, Petaling Jaya, Selangor, Malaysia; 4 Department of Oral Pathology, Lincoln University College, Petaling Jaya, Selangor, Malaysia; 5 Department of Oral and Maxillofacial Surgery, Government Dental College, Jodhpur, Rajasthan, India

**Keywords:** Head and neck squamous cell carcinoma, chemotherapy, cisplatin, immunotherapy, clinical trials, combination therapy

## Abstract

Head and neck cancer (HNC) remains a global health challenge due to its high mortality and morbidity. Advances in chemotherapy, combination therapies and targeted treatments like immunotherapy, have significantly improved survival rates. These developments pave the way for personalized therapies that maximize effectiveness while minimizing toxicities. However, challenges such as tumor resistance, treatment-related side effects and limited access to advanced therapies continue to hinder progress. Addressing these issues requires efforts in clinical research, biomarker discovery and ensuring equitable access to innovative treatments worldwide.

## Background:

Chemotherapy, from the Greek (chemo- meaning 'chemical' and -therapy meaning 'treatment'), is among other things, a chemical agent used to treat something, most often cancer [1]. Head and neck squamous cell carcinoma (HNSCC) is the sixth most commonly occurring cancer with significant treatment challenges. It is a very aggressive type of cancer, as approximately 700,000 new cases are diagnosed each year and its five-year survival rate is low (from approximately 40 to 50%) despite therapeutic advances [2]. The increased application of chemotherapy in the treatment of HNSCC, especially for loco regionally advanced cases and administered together with radiation approximately half the time, is now an established part of head and neck squamous cell carcinoma management. The introduction of molecularly targeted therapies has enhanced treatment results in recurrent or metastatic disease [3]. Environmental exposures contribute to the majority of the etiology of HNSCC, with the most potent risk factors being smoking, alcohol use and drug use. Other known risk factors are passive smoked tobacco, pollution and infectious agents. Head and neck squamous cell carcinoma is also a significant disease affecting socioeconomically disadvantaged communities where these risk factors are typically standard [4].

Age is another factor, as the average age of diagnosis is 66 years. Increasingly, HPV (human papillomavirus) is realized to play a role in, particularly in oropharyngeal cancers. Compared to HPV-negative HNSCC, which often requires aggressive therapy, HPV-positive tumors, especially those driven by genotype 16, are more responsive to treatment, including chemotherapy. With a better understanding of the molecular biology of head and neck cancer over recent decades, there has been an evolution in the chemotherapy regimens used to treat this disease in the context of both loco regional and distant disease [5]. Platinum-based medications like cisplatin (CDDP) continue to be a mainstay of therapy, usually alongside radiation [6]. Hope for improved outcomes have also emerged by incorporating targeted therapies and immunotherapy into clinical practice. Unfortunately, chemotherapy for the treatment of head and neck squamous cell carcinoma is not without pitfalls. Such are the treatment resistance, unbearable toxicity and lack of predictive biomarkers. Moreover, the molecular heterogeneity of head and neck cancer further complicates treatment strategies. Barriers such as diagnosis at a late stage, lack of healthcare access in some areas and psychosocial effects of treatment as well prevent optimal results [7, 8]. Therefore, it is of interest to review the use of chemotherapy in HNSCC, highlighting novel therapies and clinical trials of clinical relevance and the urgent need for individualization of treatment to enhanced survival and quality of life.

## Progress and advancement:

**Figure 1:**
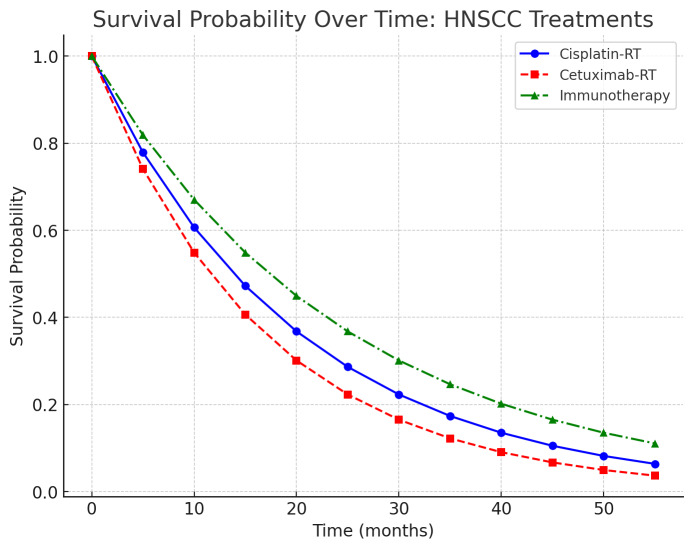
Survival Probability over Time: head and neck squamous cell carcinoma treatments

Locally advanced head and neck squamous cell carcinoma is defined as stage III or higher (T3 or higher and/or N2 or higher) per the American Joint Committee on Cancer version 7 [9]. About half of patients with PULA receive primary surgery, while the other half undergoes primary, definitive radiation therapy (RT). For patients with HPV-negative, locally advanced head and neck squamous cell carcinoma and high-risk features, recurrence rates after surgery alone are often high, requiring postoperative treatment strategies. Early approaches included adding concurrent cisplatin to postoperative RT. Adjuvant chemotherapy, particularly cisplatin-based regimens, targets micro-metastatic disease to reduce recurrence, though its role is still under investigation [10]. Two key phase III trials, RTOG 95-01 and EORTC 22931, compared postoperative RT with or without concurrent cisplatin in high-risk, HPV-negative, non-oropharyngeal tumors. Both trials showed the benefits of adding cisplatin. The EORTC trial found improved local-regional control, disease-free survival and overall survival, while the RTOG trial showed improved local-regional control but no overall survival benefit. Both used the same cisplatin schedule, but EORTC included a wider range of high-risk features, while RTOG focused on factors like metastatic lymph nodes and positive surgical margins. Despite cisplatin efficacy in controlling disease, its significant toxicities-such as microsites, dermatitis, nausea, neutropenia, kidney damage, peripheral neuropathy, tinnitus and hearing loss-have led to efforts to identify patients who benefit most from it. (Figure 1) illustrates the survival probability over time for different treatment approaches, including Cisplatin-RT, Cetuximab-RT and Immunotherapy. Immunotherapy demonstrates a better survival trend, supporting its emerging role in head and neck squamous cell carcinoma management, particularly in high-risk, HPV-negative cases undergoing chemoradiation (RTOG 0234, NRG HN003 trials) [11, 12]. A retrospective analysis of multiple trials found that extra capsular nodal extension and microscopically positive surgical margins were associated with improved disease-free and overall survival, suggesting these patients benefit more from cisplatin-based chemoradiation. However, no clear benefit was seen for those with other high-risk factors, like two or more positive nodes or perineural invasion. The analysis was underpowered, so a tiny benefit in this group cannot be ruled out. Given cisplatin's toxicity, alternative strategies are being explored. Cetuximab, an EGFR-targeting monoclonal antibody, has shown improved survival when combined with RT and is being tested postoperatively as a less toxic alternative to cisplatin-based therapies [13, 14]. In the RTOG 0234 trial, Cetuximab was combined with RT and either cisplatin or docetaxel for adjuvant treatment of high-risk head and neck squamous cell carcinoma The docetaxel-cetuximab regimen improved disease-free survival and overall survival compared to historical controls, supporting further study in HPV-negative, high-risk patients. This is being further explored in the RTOG 1216 trial, which compares cisplatin-RT with docetaxel-based regimens (docetaxel alone or with Cetuximab) to see if they offer similar or better efficacy than cisplatin-RT in high-risk HPV-negative patients. Combining immunotherapy with chemo radiation is a promising approach. The NRG HN003 trial is evaluating pembrolizumab (anti-PD1) with cisplatin-RT in high-risk, HPV-negative patients. Given HNSCC's immunosuppressive environment and PD1 upregulation after RT, pembrolizumab may enhance the immune response and improve outcomes. This phase I study assesses safety, with plans for a phase III trial comparing it to standard cisplatin-RT [15, 16].

**Figure 2:**
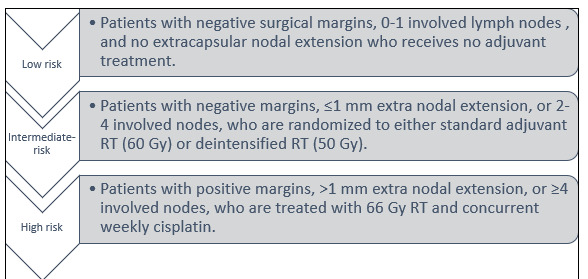
ECOG 3311 three trial risk groups

**Figure 3:**
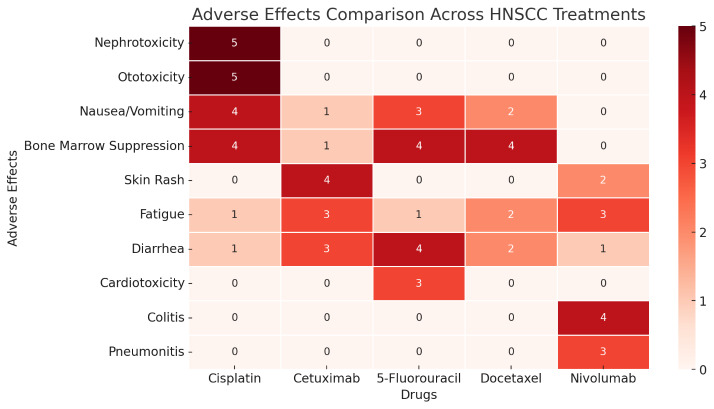
Adverse effects across head and neck squamous cell carcinoma treatments

On-going trials of docetaxel-based regimens and immunotherapy with RT are crucial for improving postoperative care for high-risk, HPV-negative head and neck squamous cell carcinoma patients, offering alternatives for those who can't tolerate cisplatin. These results will shape future treatments for this group. Adjuvant cisplatin-RT remains the standard, but outcomes for patients with perineural/vascular invasion, multiple involved nodes, or advanced T3/T4 tumors are suboptimal, often leading to postoperative RT alone. The RTOG 0920 trial tests whether adding cetuximab to RT improves outcomes for intermediate-risk patients [17]. Transoral robotic surgery (TORS) offers a less invasive option for oropharyngeal cancer, especially in HPV-positive patients. TORS improves margin-negative mucosal resections, potentially reducing the need for adjuvant chemotherapy and RT. TORS is more commonly used for HPV-positive patients, who generally have better outcomes, raising questions about whether traditional high-risk features used for HPV-negative cases should apply to HPV-positive cases. It also prompts a re-evaluation of whether HPV-positive patients could benefit from less aggressive treatment. The ECOG 3311 trial evaluates TORS combined with risk-based de-intensified adjuvant RT for clinical T1-T2, N0-N1 and HPV-associated oropharyngeal squamous cell carcinoma (OPSCC) [18]. Patients are categorized into three risk groups (Figure 2). As shown in Figure 3, cisplatin's toxicity profile includes severe nephrotoxicity, ototoxicity and bone marrow suppression, which often limits its use in certain patient populations. In contrast, cetuximab and immunotherapy agents like nivolumab exhibit different toxicity patterns, with cetuximab-associated skin rash and nivolumab-related immune-mediated effects such as colitis and pneumonitis. These variations in adverse effects play a critical role in selecting appropriate therapies based on patient tolerance and risk factors.

Since the 1980s, intensification strategies combining systemic therapy with definitive RT for unrespectable head and neck squamous cell carcinoma have been studied. A key phase III trial by the Head and Neck Intergroup showed that adding high-dose cisplatin to RT improved overall and disease-free survival, establishing cisplatin-RT as the standard for locally advanced head and neck squamous cell carcinoma [19]. However, cisplatin's high toxicity led to exploring alternatives like cetuximab, an EGFR-targeted monoclonal antibody. A phase III trial demonstrated that adding cetuximab to RT improved loco regional control and survival without significantly increasing severe side effects. Cetuximab is now a standard treatment for both HPV-positive and HPV-negative head and neck squamous cell carcinoma [20]. (Table 1) depicts an overview of various drugs used in management along with indications and side effects for head and neck squamous cell carcinoma.

In HPV-positive, undifferentiated locally advanced head and neck squamous cell carcinoma treatment has developed due to its better prognosis compared to HPV-negative cases. Cisplatin-RT has gone amp name therapy with HPV condition acting amp important Role inch endurance outcomes. Patients are classified into low-intermediate and high-risk groups based on HPV status, smoking history and nodal stage. For low-risk hpv-positive patients (t1-t3 n0-n2a), de-intensification strategies point to cut discourse strength while, maintaining remedy outcomes [21]. Chemotherapy de-intensification in HPV-positive ops focuses on the reduction of chemotherapy strength, spell maintaining efficaciousness arsenic these cancers principally answer break to discourse. The goal is to minimize treatment-related toxicities such as microsites and long-term complications while preserving cancer control. Strategies include reducing chemotherapy doses or employing radiation-sparing techniques bespoke to person diligent factors to care for HPV condition. Recent trials explore lower-dose chemotherapy radiation therapy or immunotherapy as alternatives to standard regimens. In the case of cetuximab-RT, reliable arsenic associates exist in nursing options for cisplatin-RT in low-risk patients. Spell reduced-dose radiation has shown auspicious progression-free endurance rates. However, high-risk patients may still require more intensive treatment, with on-going studies aiming to refine Rules for more personalized and less toxic options [22].

Induction chemotherapy using agents like cisplatin 5-FU and docetaxel shrinks tumors before surgery or radiation. Spell's endurance benefits bear a modest inch around trials and bespoke approaches point perspective for deficient patients. Immunotherapy is also being explored as a complement to induction chemotherapy to Improve outcomes for aggressive disease [23]. For laryngeal cancer, the focus remains on organ preservation, particularly avoiding laryngectomy. Early-stage cancers (T1-T2) often achieve high laryngectomy-free survival rates with larynx-preservation surgery or radiation. Concurrent CRT is preferred for patients with preserved laryngeal function and emerging treatments, including targeted therapies and immune checkpoint inhibitors, are under investigation to enhance efficacy while reducing toxicities [24]. Emerging therapies and ongoing research emphasize targeted therapies, immunotherapy and combination regimens for both HPV-positive and HPV-negative head and neck squamous cell carcinoma These efforts highlight the shift towards personalized treatment approaches that improve survival and quality of life. Advance in molecular profiling hold promise for developing more effective and less toxic therapies in the future [25].

## Views and opinion:

Diagnosing and treating head and neck squamous cell carcinoma (HNSCC) have shown significant advances through improved diagnostic tools, molecular characterizations and multipronged therapeutic approaches during the past ten years. The complex nature of head and neck squamous cell carcinoma requires individualized therapeutic approaches that integrate tumor biological characteristics with patient health conditions and life quality considerations. Targeted treatments combined with immunotherapy have transformed the standard treatment methods for head and neck squamous cell carcinoma According to research findings, survival rates improve following anti-PD-1/PD-L1 inhibitor use in patients with recurrent or metastatic disease. Researchers Lechner *et al.* (2022) emphasize that molecular biomarkers serve an essential role in helping doctors choose better treatments for patients with head and neck squamous cell carcinoma [5]. The treatment responses and beneficial prognoses observed in HPV-positive tumors demonstrate the critical value of molecular changes in creating personalized therapy plans [2].

HPV-associated head and neck squamous cell carcinoma de-escalation methods receive increasing attention because they decrease treatment-related side effects without compromising oncological treatment outcomes. Rosenberg and Vokes (2021) explored the scientific basis for therapeutic de-escalation by showing that reduced treatment intensity creates sustained functionality while preserving disease control [22]. Harari *et al.* (2014) demonstrate through research that risk-based classification systems using HPV status and smoking history can help guide doctors to select less aggressive treatment approaches for particular patients [15]. Radiotherapy appears as an essential therapeutic modality that head and neck squamous cell carcinoma physicians administer, most commonly with surgical interventions and chemotherapy sessions. The research of Cooper *et al.* (2012) proved that head and neck squamous cell carcinoma (HNSCC) patients with loco regional progression achieved better local control and survival results through concurrent chemoradiotherapy (CRT) approaches [12]. The management strategy produces immediate and long-term treatment side effects that include xerostomia dysphagia and microsites. The implementation of intensity-modulated radiotherapy (IMRT) techniques by Bernier *et al.* (2005) showed considerable improvement in both treatment precision and radiated structure protection [11]. Novel systemic medicine approaches show promise in boosting the performance of CRT, according to recent research findings. Research by Vermorken *et al.* (2023) analyzes how targeted drugs, including EGFR inhibitors, enhance treatment outcomes in high-risk patients [19]. Burtness *et al.* (2005) showed that combining cetuximab with radiation therapy produces promising results for improved survival rates [20]. Miranda-Galvis *et al.* (2021) identified that understanding tumor microenvironment dynamics with immune regulation determines therapeutic response patterns, particularly for patients receiving immunotherapy [4]. According to Dong *et al.* (2021), researchers demonstrated that understanding HPV-related oncogenes is formation leads to developing better, safer treatments for patients who have HPV-positive head and neck squamous cell carcinoma [21]. Sophisticated imaging techniques and augmented reality systems have proven essential diagnostic and treatment planning tools for head and neck squamous cell carcinoma (HNSCC). Augmented reality technology yields improved surgical navigation precision, according to Kashwani *et al.* (2025), leading to reduced operation times and superior surgical results [26]. Fair access remains challenging despite these accomplishments, especially for innovative medications in low- and middle-income countries. According to Sindhu *et al.* (2019), disparities exist in specialized medical services and global efforts must be launched to resolve such healthcare inequalities [9]. Research into the future of head and neck squamous cell carcinoma treatment demands the resolution of therapy complications and improved patient satisfaction measurements. Both Gougis *et al.* (2019) and Campbell *et al.* (2022) emphasize that head and neck squamous cell carcinoma patients benefit enormously from multidisciplinary care models and survival programs that fulfill their extended care requirements [8, 23]. Chemotherapy has seen significant progress in the treatment of head and neck cancer (HNC), particularly with the advent of personalized treatments, targeted therapies and the integration of immunotherapy. Recent studies highlight the effectiveness of combining traditional chemotherapy agents, like cisplatin and 5-fluorouracil, with newer immunotherapies, such as pembrolizumab and nivolumab, which enhance survival in patients with recurrent or metastatic disease [26, 27]. Moreover, developing advanced drug delivery systems has reduced toxicities, improving patient quality of life and adherence to treatment [28]. However, challenges persist, including chemotherapy resistance, as tumors exhibit significant heterogeneity and develop mechanisms to evade treatment [29]. The severe side effects of chemotherapy, such as microsites and dysphagia, also limit its effectiveness and impact long-term recovery [30]. Late-stage diagnosis further complicates treatment, as patients often present when the disease is more challenging to treat [31]. Additionally, the high costs associated with newer therapies pose barriers to widespread access, particularly in low-resource settings [32]. Overall, while advancements in chemotherapy have improved outcomes, overcoming resistance, managing side effects, early detection and ensuring access to treatment remain critical for maximizing its potential.

## Future directions and perspective:

The management of head and neck squamous cell carcinoma (HNSCC) is set to undergo significant changes through the incorporation of advanced technologies such as artificial intelligence (AI), the metaverse, virtual reality (VR) and augmented reality (AR) [33, 34]. AI-driven solutions will facilitate early identification, customized treatment planning and toxicity forecasting by analyzing intricate imaging, pathology and genome datasets. The metaverse has the potential to transform patient treatment through the establishment of virtual tumor boards for international collaboration, immersive patient education and interactive rehabilitation programs [35]. Virtual reality can potentially improve surgical training and preoperative planning and alleviate patient anxiety, whilst augmented reality may facilitate precise treatment via guided surgical navigation and enhanced radiotherapy administration. Synergistic applications, including AI-driven virtual reality simulations and metaverse-integrated AR platforms, are expected to strengthen collaboration, precision and patient involvement [36]. Notwithstanding problems with data security, accessibility and ethical considerations, these technologies provide a means to provide more accurate, effective and patient-centered therapy, transforming the future of oncology and enhancing results for head and neck squamous cell carcinoma patients.

## Conclusion:

The progress in chemotherapy, encompassing cisplatin-based protocols, targeted treatments and immunotherapy has markedly enhanced outcomes for patients with head and neck cancer, particularly in high-risk populations. Notwithstanding these advancements, issues such as treatment toxicity, tumor resistance and restricted access to modern medicines endure, requiring continuous clinical research and equitable healthcare measures. Customized strategies and novel treatments are poised further to improve these patients' survival and quality of life.

## Figures and Tables

**Table 1:** Overview of treatment mechanisms, side effects and indications for HNSCC

**Drug**	**Mechanism of action**	**Key toxicities**	**Indications**
Cisplatin	A chemotherapy drug that forms a charged platinum complex inside cells, which binds to and disrupts DNA, stopping cell growth and division.	-Nephrotoxicity	-Primary treatment for advanced head and neck squamous cell carcinoma with radiation
		-Ototoxicity	-Post-surgery adjuvant treatment
		-Nausea/vomiting	-Induction chemotherapy
		-Nerve damage	-Metastatic/recurrent HNSCC
		-Bone marrow suppression	
Cetuximab	A monoclonal antibody that targets and blocks the EGFR on cancer cells, preventing them from growing.	-Acne-like skin rash	-Primary treatment for locally advanced head and neck squamous cell carcinoma with radiation
		-Fatigue	- Post-surgery adjuvant treatment
		-Diarrhea	-Metastatic/recurrent HNSCC
		-Low Hypomagnesemia	
5-Fluorouracil	An antimetabolite that interferes with DNA and RNA synthesis by blocking thymidine production, halting cell division.	-Alopecia	-Induction chemotherapy
		-Low blood counts (Bone marrow suppression)	-Metastatic/recurrent HNSCC
		-Diarrhea	
		-Heart damage (Cardiotoxicity)	
Docetaxel	A chemotherapy drug that binds to microtubules, preventing cells from dividing by blocking DNA, RNA and protein synthesis.	-Fluid retention	-Induction chemotherapy
		-Alopecia	-Metastatic/recurrent HNSCC
		-Low blood counts (Bone marrow suppression)	
		-Stomatitis	
Nivolumab	An immunotherapy drug that blocks PD-1 on T-cells, enabling the immune system to attack cancer cells more effectively.	-Colitis	-Metastatic/recurrent HNSCC
		-Pneumonitis)	
		-Dermatitis	
		-Hepatitis	
